# A50 PRODUCTION OF A PANCREATIC AND INTESTINAL HEPATOCYTE NUCLEAR FACTOR 4A DOUBLE KNOCKOUT MOUSE MODEL TO UNRAVEL NEW MECHANISMS CONTROLLING METABOLISM

**DOI:** 10.1093/jcag/gwad061.050

**Published:** 2024-02-14

**Authors:** N Rodriguez Rodriguez, A Di Castro, C Jones, D Pupo Gómez, F Boudreau

**Affiliations:** Immunology and Cell Biology, Universite de Sherbrooke Faculte de Medecine et des Sciences de la Sante, Sherbrooke, QC, Canada; Immunology and Cell Biology, Universite de Sherbrooke Faculte de Medecine et des Sciences de la Sante, Sherbrooke, QC, Canada; Immunology and Cell Biology, Universite de Sherbrooke Faculte de Medecine et des Sciences de la Sante, Sherbrooke, QC, Canada; Immunology and Cell Biology, Universite de Sherbrooke Faculte de Medecine et des Sciences de la Sante, Sherbrooke, QC, Canada; Immunology and Cell Biology, Universite de Sherbrooke Faculte de Medecine et des Sciences de la Sante, Sherbrooke, QC, Canada

## Abstract

**Background:**

Hepatocyte nuclear factor (HNF) 4A is a transcription factor mainly found within endodermal organs, including the intestine and pancreas. HNF4A controls the expression of genes associated with glucose transit, glycolytic enzymes, lipid and drug metabolism, and incretin production from intestinal enteroendocrine cells. Mutations within this are linked to various diseases, including maturity-onset diabetes of the young and increased risk of Type 2 diabetes mellitus. However, it remains unclear whether intestinal and pancreatic HNF4A is functionally involved in glucose metabolism. This could be partly due to the lack of suitable experimental model that allow recreating phenotypes associated with *hnf4a* lost in two key organs involved in controlling body glucose levels.

**Aims:**

To generate a conditionally mutant mouse line that does not express HNF4A in the intestine or the pancreas and to investigate how glucose metabolism is affected in these mice.

**Methods:**

Conditional deletion in each organ was achieved by mating *VillinCre* or *InsCreER* with *Hnf4α*^loxP/loxP^ C57BL/6 mice, respectively. The mutant mouse line was then generated by crossing mice *Hnf4α*^loxP/loxP^*+VillinCre* and *Hnf4α*^loxP/loxP^+*InsCreER*. PCR was performed to confirm all genotypes. Male and female mice around 2-3 months were distributed in four groups, including controls, *Hnf4α*-deleted intestine only, *Hnf4α*-deleted pancreas only, and *Hnf4α*-deleted in both organs. HNF4A expression was measured by qPCR, immunoblot and immunofluorescence. Glucose metabolism tests, including oral glucose tolerance (2g/kg) and insulin tolerance (0.75 International Units/kg) were performed. The blood glucose levels were measured after overnight fasting at 15, 30, 60, and 120 min.

**Results:**

The body weight of four groups of mice remained comparable over experimental time. HNF4A expression was not detected from double mutant mice’s gut epithelial or pancreatic β cells. The oral glucose challenge showed a difference among the group of experimental mice as compared to the control group. After 15 min, blood glucose values of double mutant mice remained higher than control mice, for which glucose clearance was less efficient in the mutants. Insulin tolerance tests demonstrated that double mutant mice behave differently than control mice, suggesting a defect in insulin response.

**Conclusions:**

These promising preliminary results allow us to obtain and characterize a novel murine model to study glucose metabolic syndrome that could be related to *hnf4a* gene deletion in both the gut and pancreas. Further studies are needed to establish a mechanism by which the HNF4A transcription factor could interplay between the intestine and the pancreas during whole-body glucose metabolism.

**Funding Agencies:**

CIHRBourses d’excellence de l'Université de Sherbrooke

